# Mental Health and Mental Wellbeing Impact Assessment Frameworks—A Systematic Review

**DOI:** 10.3390/ijerph192113985

**Published:** 2022-10-27

**Authors:** Johanna Cresswell-Smith, Tapani Kauppinen, Taina Laaksoharju, Tuulia Rotko, Pia Solin, Jaana Suvisaari, Kristian Wahlbeck, Nina Tamminen

**Affiliations:** 1 Finnish Institute for Health and Welfare, 00271 Helsinki, Finland; 2 Mieli—Mental Health Finland, 00240 Helsinki, Finland

**Keywords:** mental health, mental wellbeing, impact assessment, mental health promotion

## Abstract

Mental health is largely shaped by the daily environments in which people live their lives, with positive components of mental health emphasising the importance of feeling good and functioning effectively. Promoting mental health relies on broad-based actions over multiple sectors, which can be difficult to measure. Different types of Impact Assessment (IA) frameworks allow for a structured approach to evaluating policy actions on different levels. A systematic review was performed exploring existing IA frameworks relating to mental health and mental wellbeing and how they have been used. A total of 145 records were identified from the databases, from which 9 articles were included in the review, with a further 6 studies included via reference list and citation chaining. Five different mental-health-related IA frameworks were found to be implemented in a variety of settings, mostly in relation to evaluating community actions. A Narrative Synthesis summarised key themes from the 15 included articles. Findings highlight the need for participatory approaches in IA, which have the dual purpose of informing the IA evaluation and advocating for the need to include mental health in policy development. However, it is important to ensure that IA frameworks are user-friendly, designed to be used by laypeople in a variety of sectors and that IA frameworks are operational in terms of time and monetary resources.

## 1. Introduction

Mental health is largely shaped by the social, economic and physical environments in which people live their lives [1,2]. This finding has contributed towards a growing shift from viewing mental health as purely an individual issue to be addressed by the healthcare sector, to one which is of relevance throughout all sectors of society [3,4,5]. Another shift in attention is towards positive components of mental health which represents an approach which not only focuses on the treatment or prevention of mental health disorders, but also emphasises the importance of feeling good and functioning effectively [6]. The World Health Organisation formulated the well-cited definition of (positive) mental health as “A state of well-being in which every individual realises his or her own potential, can cope with the normal stresses of life, can work productively and fruitfully, and is able to make a contribution to her or his community” [7]. Although arguably culturally determined [8], this definition moves away from a clinical definition of mental health and emphasises the importance of day-to-day environments. Positive mental health can be promoted at the structural, community and individual level [9]. On an individual level, evaluating the impact or effectiveness of positive mental health interventions can be measured using measures such as the WHO-5 [10] or the Warwick-Edinburgh Mental Well-Being Scale (WEMWBS) [11]. Evaluating the impact of community-based actions however, for example, access to leisure activities, closing down a primary school or changes to national policy, can be more difficult to measure.

With origins stemming from Environmental Impact Assessment, Impact Assessment (IA) approaches can be defined as “the process of identifying the future consequences of a current or proposed action with the ‘impact’ being the difference between what would happen with the action and what would happen without it” [12]. Although it is often not possible to gauge this difference in an exact or linear manner, attempts have been made to systematically approach this complex relationship. For example, the Health Impact Assessment (HIA) is a structured, stepwise process used to evaluate health-related impacts of decisions based on participatory processes [13,14,15,16]. Despite HIA practice having diversified throughout the past twenty years, lack of awareness and capacity in terms of their use continues to contribute towards innovative actions going unnoticed [16]. Offshoots of the HIA include IA frameworks focusing specifically on mental health impacts. Despite the importance of mental health on population health [17], mental health IA has received comparatively less attention than other forms of IA such HIA or Environmental Impact Assessment (EIA) [16]. Although the diversity of different IA frameworks has been reported as a possible source of confusion, other approaches advocate for the need for specific tailor-made frameworks to increase both interest and adherence [16]. In order for IA frameworks to be used in policy making they need to be easily available and clear. Chanchitpricha and Bond [18] outline effectiveness in relation to different IA frameworks in terms of (a) “procedural effectiveness” relating to practice and whether there is clarity why and how IA is undertaken, (b) “substantive effectiveness” relating to performance and whether IA is integrated into decision-making, and whether objectives are met, (c) “transactive effectiveness” relating to proficiency and if resources are managed efficiency and (d) “normative effectiveness” relating to purpose and whether goals are achieved. Although this framework is in its infancy, it provides some guidelines for exploring suitability of different IA frameworks.

There appears to be a dearth of information in terms of the availability of mental-health-related IA tools. The objective of the current study is to systematically review existing IA frameworks relating to mental health and mental wellbeing impacts, exploring what types of IA frameworks have been used, and to what extent in all age groups, countries and contexts. Throughout this article, ‘mental health IA’ is used as an umbrella term to denote IA approaches which include a general focus on mental health, including both continuums ranging from mental health disorders and difficulties to mental wellbeing [19]. When specific components of this umbrella term (i.e., mental health disorders or mental wellbeing) are discussed, they will be defined separately.

## 2. Materials and Methods

A search was conducted for studies with a clear reference to mental health or mental wellbeing impact assessment frameworks, guided by a published protocol registered on the PROSPERO website (registration code: 324895).

### 2.1. Search Strategy and Eligibility Criteria

Searching began in March 2022 and ended in June 2022, including published literature in all languages. Databases included EDS, Scopus, ERIC, PsycINFO, CINAHL, SocIndex with fulltext. Search terms relating to mental health or mental wellbeing impact were combined with proximity operators within five words denoting a model, evaluation, or framework. Truncation was used in order to maximise reach. Search procedures followed PRESS guidelines [20]:

(mental health impact assessment” OR “mental health impact” N5 (model* OR evaluat* OR valuat* OR framework))

(“mental wellbeing impact assessment” OR “mental wellbeing impact” N5 (model* OR evaluat* OR valuat* OR framework))

(“mental well-being impact assessment” OR “mental well-being impact” N5 (model* OR evaluat* OR valuat* OR framework))

Furthermore, the reference lists of all eligible studies and relevant review articles were reviewed manually for additional relevant studies.

### 2.2. Inclusion and Exclusion Criteria

The systematic literature review includes all quantitative and qualitative study designs which use a specific framework or model striving to evaluate the impact of a population-based action on mental health or mental wellbeing. Studies measuring mental health impact on a strictly individual level were omitted, for example, those which evaluated “mental health impact of cognitive behavioural therapy on depression”. Examples of inclusion and exclusion details are outlined in Table 1.

### 2.3. Study Identification

JCS conducted the search process and search results were saved into the Zotero reference management system, duplicates were removed, and the resulting articles were imported into the Rayyan QCRI software to facilitate the study selection process.

JCS reviewed all articles on title level tagging articles into preliminary ‘included’, ‘excluded’ and ‘maybe’ categories. PS and NT then reviewed the selections, and any queries or uncertainties were resolved via discussion. No discrepancies in the selection process arose. Backward and forward citation chaining of all included articles were conducted to identify further studies meeting the search criteria.

### 2.4. Data Presentation, Analysis and Synthesis

Data was extracted into an Excel spreadsheet under the following headings: (1) authors, (2) year, (3) country, (4) evaluator level, (5) funder, (6) setting and participants, (7) procedure (why and how), (8) objective(s), (9) proficiency of process (time, finances, skill, roles) and (10) purpose (what goals were attained), see Table 2 and Table 3. The topic was not expected to result in a large number of studies; therefore, a Narrative Synthesis denoting observations and common themes was used to summarise findings [21].

No suitable quality assessment framework was found for studies on IA. Effectiveness was therefore explored using a mapping exercise inspired by a recent approach outlined by Chachitpricha & Bond [18] including the following four categories: (1) Practice, that is, what procedures/principles were used, (2) Performance, in terms of how objectives were met, (3) Proficiency as in how resources were managed and (4) Purpose in terms of what goals were attained.

## 3. Results

### Included Studies and Study Characteristics

**Figure 1:**
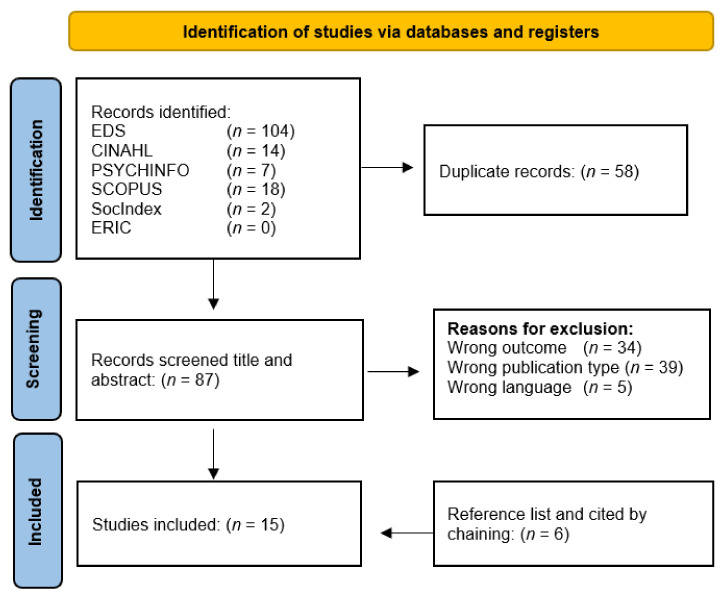
Flow diagram of article selection process.

A total of 145 records were identified from the six databases. After the removal of duplicates, 87 studies were reviewed for eligibility on title and abstract assessment, after which a further of 77 records were omitted. Primary reasons for exclusion were “wrong outcome” (*n* = 34) denoting records which may have had the correct order of wording in accordance with the search terms, but on further reading the focus was not in-line with the current objectives. For example, studies assessing the “mental health impact” of a particular treatment or intervention on an individual level. Another leading reason for exclusion was “wrong publication type” (*n* = 39), reflecting articles which may have made references to suitable impact assessment frameworks, however, were found to be magazine or newspaper entries or editorials without suitable content. Records which sounded promising in terms of their focus on the correct types of impact assessment but did not outline any actual frameworks were also included in this category. Following this process, ten articles were identified for inclusion. Further reference chaining of the included studies resulted in six further articles to be included for review. The study selection process is illustrated by the flow diagram in Figure 1.

As summarised in Table 2, five records were academic studies, four were collaborative studies including community organisations or statutory services and five were local evaluations, with one being a toolkit development paper. Table 3 collates information in terms of Chanchitpricha and Bond’s [18] framework for assessing effectiveness; that is, procedure, objective, proficiency and purpose of the included impact assessment frameworks.

## 4. Discussion

The current review summarises existing types of IA frameworks with a focus on mental health or mental wellbeing outlines how they have been used to date. A total of five different mental-health/mental-wellbeing-related IA frameworks were found. Two of these frameworks, i.e., the MWIA and MHIA had similar approaches both with roots in HIA and were developed by two different institutions. The MWIA was developed by the South London and Maudsley NHS Foundation Trust and partners (MWIA National Collaborative UK) focusing predominantly on positive definitions of mental wellbeing although does include references to negative impacts such as mental health difficulties. The MHIA on the other hand was developed by the Institute on Social Exclusion (ISE) at the Adler School of Professional Psychology Chicago, USA, and has a stronger focus on impacts relating to mental ill health. Due to the heterogeneity of the approaches used in the included studies, it is not possible to make exact comparisons or make clear judgements in terms of effectiveness. However, it is possible to report on the context in which they have been used and describe observation in terms of approach, ethos and effectiveness (see Table 3).

Just over half (9/15) of the included studies used their IA frameworks in a similar context, that is, assessing the impact of population-based interventions or policies on deprived communities, with seven using the MWIA and the remaining two using the MHIA. The included studies predominantly focused their attention on reporting actual findings from their impact assessment process, although it was possible to pick out certain evaluative details in terms of usefulness of the actual IA framework in their particular context.

### 4.1. Impact Assessment of Community Projects

The report by Holmes et al. [24] was a summary of five community projects aiming to improve the wellbeing of people living within the most disadvantaged communities in the north-west of the UK. The MWIA framework was used in a similar manner in all projects, including participative workshops and community profiles. The benefits of the MWIA process included appreciation for the community workshops and the opportunity to discuss and define mental wellbeing. The approach was similar to that of Shearn et al. [27] who reviewed the Art into Life community project in London. The benefits of the impact assessment included increased confidence in talking about mental wellbeing, increased insights into ones’ own mental wellbeing, and an appreciation for the practical information-gathering approach were reported [27]. The importance of careful adaptation was highlighted, and a concern that the use of ‘jargon’ was a challenge to some stakeholder groups, in this case older adults with dementia and mental and physical health problems. Edmonds [25] used the MWIA framework to assess impacts of the Well London MWIA Training and Capacity Building programme. The report outlined important challenges in implementing the MWIA including lack of stakeholder engagement, identification of suitable projects, disconnect due to staff turnover. It also reported difficulties in investing in the MWIA process, particularly for projects with limited resources as the MWIA practitioners were external to the projects they were assessing. On the positive side, however, training in the MWIA framework was appreciated, especially as it brought people together from different London Boroughs forging new partnerships. Additionally, the structure and the resources of the provided framework was valued. West et al. [26] used the MWIA to assess impacts of the Liverpool 2008 European Capital of Culture programme on mental wellbeing, screening through a total of 16 projects and policies. The study used a wide variety of data sources stemming from stakeholder workshops, literature review and community profiles, triangulating these to produce a rich understanding. The MWIA process was reported to raise awareness about mental wellbeing among project leads and stakeholders and have a positive effect on networking and collaboration. However, challenges such as engaging the arts and business sectors, the need for simplified terminology and ensuring clarity for all participants was also pinpointed. Stewart et al. [35] used the MWIA framework, in addition to the Wheel of Wellbeing, to assess regional Mental Health and Wellbeing Hubs in Queensland revolved around raising awareness (of positive mental health), capacity building and coordination activities (of networks and local stakeholders). This in-depth evaluation process included focus groups as well as SWOT analyses and a document and literature review. The resulting summary was well-structured and the evaluation process itself reportedly adding value to the formation and strength of collaboration within the Wellbeing Hubs. The MWIA was used to empower local engagement and planning processes while providing structure for both evaluation and advocacy. The report did not detail specific details about how the MWIA was used, although several references were made to it and the resulting report displayed a good level of detail around many of the themes included in the MWIA toolkit [28].

Krasner & Copeland [22] describe a community renewal scheme within the New Deal for Communities [37] scheme in an area of deprivation in Hull, UK. The assessment process developed by the initiative, had synergies with the MWIA framework, including stakeholder workshops, and a focus on participation, inclusion and community safety for indicator development. It also recognised mental health impacting individual, community and structural levels. The approach took a positive view of mental health but stated that it was “difficult for many people to comprehend [positive mental health] as they perceive mental health to be a negative term, reflecting a problem” [22]. This may reflect the period during which the report was written, 2003 when positive approaches to mental health were still under development. The evaluation process itself was described as lengthy, taking place alongside other tasks, with the end-result reported to be updated at a later date. Authors were not able to locate any further literature pertaining to this final report.

### 4.2. MWIA as an Outcome Measure or Study Evaluation Tool

In an intervention study Chiumento et al. [34] adapted the MWIA to evaluate the Haven Green space intervention for children experiencing behavioural, emotional and social difficulties in three schools in the north-west of England. The MWIA was used to assess the study’s primary outcome described as children’s wellbeing along with some secondary measures. The MWIA toolkit was used to (1) define wellbeing and (2) plot protective factors ‘enhancing control’, ‘increasing resilience and community assets’ and ‘participation and social inclusion and was conducted pre- and post-intervention. Despite the approach being age-adapted, authors questioned how some of the “adult terminology” was to be understood by children. Despite these limitations, the MWIA was reported to have clearly resonated with children, and it was recommended for use following further age-appropriate adaptation. This study clearly used the MWIA in a different manner to others using the same framework. While it was mentioned that the MWIA was used as the qualitative component in the mixed methods study, it appeared to be used more as a pre- and post-intervention study measure.

Burford et al. [33] presents two case studies using the MWIA in two different workplace contexts, including screening, stakeholder interviews and workshops. MWIA’s were facilitated using external facilitators which was reported to aid coordination as well as providing a safe space for the participants to freely voice their concerns that may affect their workplace wellbeing. In both case studies, the MWIA process increased the understanding and awareness of mental wellbeing. The authors also brought up the dual purpose of the MWIA acting as a vehicle for change as well as being an evaluative tool [33]. Engagement with senior management was identified as important for successful outcomes in both case studies, although many of the actions placed emphasis what the team and individuals can do to improve their own wellbeing. Participants felt that the MWIA workshop gave them the opportunity to have their voice heard by senior management and that they were allowed to express concerns. Furthermore, it helped team members appreciate issues that others within the team were facing, such as age, circumstances in their personal life and career aspirations and ambitions. How to ensure that actions are actually implemented was articulated as a challenge, emphasising the need for a follow up component.

### 4.3. Mental Health Impact Assessment: Focus on Ill-Health

Todman et al. [30] describe a pilot study set in Chicago’s Englewood community using the MHIA framework in relation to an amendment in the Vacant Buildings Ordinance. Assessment included a comprehensive process including screening, scoping, assessment, recommendations and included active engagement of community residents, resulting in a series of pathway diagrams denoting hypothesised connections between the amendment and mental health in the targeted community. Mental health impacts centred on illness or distress, including mental health outcomes such as depression, anxiety, symptoms of post-traumatic stress disorder (PTSD) and attention deficit/hyperactivity disorder (ADHD) and substance abuse, rather than positively slanted outcomes. McDowell et al. [31] worked with the same community, investigating whether a policy change relating to conviction records in employment decisions impacts individual and community-level mental health outcomes. The authors describe the study setting in detail, acknowledging the impact of the process itself and the need for empowering authentic partnership working [31]. Interestingly, the paper does not detail actual results of the MHIA in detail (i.e., how the policy change impacted mental health outcomes) notwithstanding a pathway diagram depicting relationships between policy change, social determinants and mental health. Instead, the article focused on reporting on the process of the IA. This approach is particularly interesting as it underlines the need for genuine partnerships and the importance of developing long-standing relationships within a community, in order to be able to build capacity around meaningful and shared goals.

### 4.4. Mental Health Impact Assessment of Societal Crises

A total of three articles assessed the mental health impact of a societal crises on a population. Palinkas [29] developed a conceptual framework for understanding and responding to the social and psychological impacts of the Deepwater Horizon oil spill. The resulting conceptual map builds on three theoretic traditions to produce three interlinked tiers of impact—Environmental, Community and Intrapersonal—all containing further subcategories. The conceptual framework was then tested using data from a previous oil spill producing promising results in terms of the frameworks useability; however, it was acknowledged that a comprehensive examination was not possible. The conceptual model offered a strategy for organising evidence but recognised the need for longitudinal data. Although qualitative interviews were performed with community stakeholders during the development process, Palinkas et al. [29] placed more emphasis on the need for the following quantitative indicators (among others) for longitudinal assessment: changes in employment, income and cultural activities, short-term and long-term health status, measures of social support, prevalence of mental disorders, stress-related illnesses, domestic violence, drug and alcohol abuse, child behavioural problems. There was no focus on positive mental health included in the study. Ampuero et al. [32] on the other hand used the MWIA to identify and describe factors enhancing mental wellbeing of people affected by a tsunami on Robinson Crusoe Island in 2010 and explores their effects on people’s resilience. The MWIA framework was used to (1) identify and describe the main factors enhancing mental wellbeing of people affected by a natural disaster, (2) understand the environment through which influencing factors affect mental wellbeing, (3) describe the environmental dynamics of influential factors. Qualitative data was gathered including interviews, fieldwork observation and artefacts analysis to produce an environmental model based on aspects of the natural environment, meaningful activities, local food, social activities, lifelong learning, transport and security influencing mental wellbeing through four main environmental dimensions: ecology, culture, milieu and organisation. The study pinpointed the lack of attention to the cumulative effect of different extrinsic factors as a limitation of the MWIA. Finally, the recent paper by Lin et al. [36] focused on the impact of the COVID-19 pandemic on adolescents, to develop an ecological framework including four domains of factors (school, family, healthcare system, community) which were considered to directly or indirectly impact the mental health of children and adolescents during the pandemic. The four factors influence on mental health was considered on the micro (e.g., family members and peers) meso (e.g., interplay between school/family/healthcare providers), exo (e.g., parents’ experience) and macro (e.g., economic condition of the society) systems, providing a theoretical foundation for informing researchers and clinicians of how to evaluate mental health issues among children and adolescents in the event of a future pandemic. The ecological framework was reported to be useful for expanding the scope of research questions and encouraging cross-disciplinary connections, as well as help clinicians identify predisposing, precipitating and perpetuating factors, associated with the pandemic. Although this framework did include a focus on clinical uses (e.g., informing intervention planning), it also identified stressors within the ecosystem requiring broader cross-disciplinary strategies governed by integrative policies, making it interesting for the current review.

### 4.5. Quality Assessment

As can be seen in Table 3, it was possible to extract data from the included studies under all of Chanchitpricha and Bond’s [18] four categories, suggesting that all included studies provided a comprehensive level of information. Although effectiveness cannot be assessed in an empirical sense, Chanchitpricha and Bond [18] take steps toward establishing what criteria are important to include in IA frameworks. They also remind us of the existence of a wide variety of perspectives for ascertaining ‘effectiveness’ such as in terms of usefulness and relevance. For example, the HIA has been assessed in terms of its contributions to policymaking [38,39], the same principles can be adapted to IA for mental health. Both the MWIA and the MHIA have been applauded for their dual-purpose nature, both in terms of facilitating evaluation but also as a tool for advocacy, raising awareness and capacity building. This makes them both ideal vehicles for making contributions to policy making.

This can be argued to be especially true for the MWIA considering its focus on mental wellbeing which may reflect a contemporary paradigm shift acknowledging the benefits positive mental health for a populations’ [40]. Implementing IA for mental health is an important step towards a Mental Health in All Policies (MHiAP) approach. MHiAP highlights the need for mental health to be part of all sectors in order to develop mentally healthy societies [41] via relationship building, inter-sector collaboration, training and funding mechanisms [42,43].

### 4.6. Strengths and Limitations

The current review offers a unique summary of existing IA for mental health, detailing how they have been used as well as pinpointing benefits and challenges of their use. Unfortunately, the review remains on a descriptive level as the wide variety of approaches and levels of reporting makes concrete comparison difficult. It is important to consider some methodological strengths and limitations.

It was deemed important to keep the search strategy broad but still including clear wordings to keep focus on IA of population-based actions, rather than impact of individual level interventions. As there remains considerable heterogeneity in terms of IA for mental health, it is entirely possible that the search strategy omitted some articles which did not meet the current search strategy’s criteria. Authors are aware of drawbacks of using academic databases, although EBSCO database was used as it also includes grey literature. Local-level, non-published IA therefore could have been missed, for example, Holmes’ mention in their report [24] (p. 26) that there have been at least 300 Rapid MWIAs undertaken over the last five years in England; these rapid MWIA were not found by the current search strategy.

Results indicate that most included studies used the MWIA framework and were UK based. This could be an indication of a general capacity building drive towards using the toolkit among different regions and boroughs in the UK, many of the reports included involvement of statutory services or council involvement. Many of articles including the MWIA reported findings in a similar fashion which can be assumed to be related to synergies stemming from the comprehensive theoretical material and templates included in the toolkit. Furthermore, many of these reports were funded by statutory organisation either completely or in collaboration with other funders such as the National Lottery. Additionally, some academic institutions were included in the reports. Funders and partnership working with academic institutions influence how available reports are for systematic review. Reports written by academic institutions are more likely to be in a format suitable for publication in academic journals and thereby found via database searching. However, this does not mean that these are the only IA available. Funding and level of reporting has repercussions in terms of sustainability.

### 4.7. Areas for Further Development

Assessing the availability and use of existing mental health impact assessment is a timely endeavour. A recent Finnish survey appears to suggest that despite the availability of existing frameworks, along with an interest in mental health impact assessment, municipalities have difficulty implementing mental health or mental wellbeing impact assessment procedures [44]. This lines up with a recent protocol paper which also implies increased interest in including mental health impact assessment [45], a promising area of future study. It also parallels findings in terms of HIA’s being broadly used in a variety of diverse settings although publications reporting such results appear to be limited [46]. There appeared to be a general preference for rapid process HIA with subsequent reports lacking in-depth details and documentation of each stage in the process as well as justification as to why certain decisions were made [46]. Developing easily accessible approaches for mental health assessment should make use of previous experiences such as those described in the current review.

## 5. Conclusions

This systematic review details how mental health IA has been assessed in different circumstances. Developing accessible approaches to mental health IA is timely considering the increased understanding of the influence of the daily environment on a population’s mental health. Mental health IA frameworks offer concrete guidance on how to assess the impact of broad actions. Findings from studies included in the current review highlight the need for including participatory approaches, which have the dual purpose of informing the IA evaluation but also advocating for the need for attending to mental health. This is particularly true in terms of the MWIA which has the additional purpose of highlighting the need for increased attention on positive aspects of mental health. Teasing out what upholds resilience and supports coping can be an important endeavour even in relation to critical times.

Building mentally healthy environments depends on decision makers having effective and accessible evaluation tools. It is important to ensure that IA frameworks are user-friendly and designed to be used by laypeople in different sectors. Avoiding jargon and unnecessarily complicated terminology was highlighted in several of the included studies. IA frameworks also need to be useable in terms of time and monetary resources. Considering many of the included studies were at least partially funded by external grants such as Kellogg’s or the National Lottery, it is clear that these processes were not (at least at the time of writing) not fixed within statutory or municipal funding mechanisms although many did also list statutory agencies as collaborators. Resource allocation is an essential component for IA to become part of common praxis.

## Figures and Tables

**Table 1:** Examples of inclusion and exclusion criteria.

Study Focus	Included	Excluded
All population groups (i.e., all ages)	x	
English, Swedish or Finnish language	x	
Mental health or mental wellbeing impact assessment in all contexts	x	
Reports or books outlining specific frameworks for mental health or mental wellbeing impact assessment	x	
Any impact assessments which include mental health components	x	
Mental health/mental wellbeing impact assessment on individual level		x
Reports or books outlining general approach or need with no outline of specific framework.		x

**Table 2:** Included MHIA’s.

Authors	Year	Country	Evaluator Level	Funder	Setting and Participants
Krasner et al. [22]	2004	UK	Local evaluation	Government funding under New Deal for Communities initiative.	An inner-city deprived community in Hull: high unemployment, lower than average wages, low educational attainment, high turnover of residents, many boarded up properties and general evidence of environmental neglect.
Cooke & Coggins [23]	2005	UK	Academic, collaboration with statutory agency	Local Government under Lewisham Neighbourhood Renewal Fund (LNRF).	MWIA tool development process description within the setting of the Lewisham Neighbourhood Renewal Fund regeneration programmes on mental health and wellbeing.
Holmes et al. [24]	2009	UK	Local evaluation	Evaluation commissioned by Liverpool Primary Trust. Projects funded by National Lottery UK	Participants from six community projects within Liverpool Primary Care Trust targeting asylum seekers, older adults and people with existing health problems, children and young people. One community project included a broader population approach.
Edmonds [25]	2009	UK	Local evaluation	The National Lottery UK	A London-based capacity building project with a total of 93 participants including representatives form Primary Care Trusts in London local authorities, representatives of the NHS and other community organisations.
West & Scott-Samuel A. [26]	2010	UK	Academic, collaboration with statutory agency	Care Services Improvement Partnership, Liverpool Culture Company, Liverpool Primary Care Trust, Mental Health Foundation	Evaluation included 16 projects with participants representing a variety of stakeholders including arts organisations, the Culture Company, neighbourhood management services and the community members.
Shearn [27]	2011	UK	Local evaluation	Partnership between the Tate Modern and South London and Maudsley NHS Foundation Trust.	Older adults aged 60–80 with dementia and mental and physical health problems from Southwark and Lambeth which are areas of extreme contrasts ranging from tourism, culture and wealth and also high levels of social deprivation, disadvantage and mental health need.
Cooke et al. [28]	2011	UK	Toolkit	Former National Mental Health Development Unit; South London and Maudsley NHS Foundation Trust; Inukshuk Consultancy	Setting not specified—MWIA toolkit outline
Palinkas [29]	2012	US	Academic	Not reported	The conceptual framework collates previous literature describing the environmental, community and intrapersonal effects of oil spills set in the context of the 1989 Exxon Valdez oil spill in Alaska.
Todman et al. [30]	2012	US	Academic, collaboration with statutory agency	Not reported	Community residents in area of deprivation with high rates of crime and violence, underperforming schools, depressed housing stock, joblessness and few public services and amenities. It is also vulnerable to broader challenges such as racism, sexism and social exclusion.
McDowell et al. [31]	2014	US	Academic, collaboration with statutory agency	Robert Wood Johnson Foundation, W.K. Kellogg Foundation, and Pierce Family Foundation	Community residents in area of deprivation with high rates of crime and violence, underperforming schools, depressed housing stock, joblessness and few public services and amenities. It is also vulnerable to broader challenges such as racism, sexism and social exclusion.
Ampuero et al. [32]	2015	Chile	Academic	Effective Sustainable Solutions Ltd.	A total of 50 residents from Robinson Crusoe Island off the coast of Chile, participated in exploring the effects of a tsunami following an earthquake. The town was under an intensive programme of reconstruction at the time of the interviews.
Burford et al. [33]	2017	UK	Academic	Not reported	Employees from two organisations: an academic health sciences collaboration centre (including clinical services, academic research and human resources) and a local authority.
Chiumento et al. [34]	2018	UK	Academic	Primary Care Trust (now a Clinical Commissioning Group)	A total of 36 primary school pupils, aged 9–15 from two primary schools located in areas suffering from high levels of deprivation including child poverty.
Stewart et al. [35]	2019	AUS	Local evaluation	QMHC commissioned Griffith University to evaluate	Mental Health and Wellbeing Hubs located in different communities in Queensland including: (a) large, metropolitan, multicultural community, low socio-economic status; (b) rural and remote communities with lack of formal support services; (c) farming community combined with tourism and mining, unemployment, aging population, limited economic investment, limited sport and recreation activities relocating families.
Lin et al. [36]	2021	US, CAN, AUS	Academic	Not reported	Previous literature was collated to explore an ecological framework of mental health stressors stemming from the COVID-19 pandemic and anti-contagion policies on children and adolescents. Examples were compiled from different sectors (1) health care (2) community support (3) family support.

**Table 3:** Characteristics of included Impact Assessments (effectiveness).

Authors	Procedure	Objective(s)	Proficiency of Process	Purpose
Krasner et al. [22]	Framework development to evaluate community mental health using unnamed community wellbeing framework	To describe a development process for a monitoring and evaluation framework evaluating community mental health on the Preston Road Estate against a set of indicators of positive community mental health.	Local indicators used, local community engagement. Approach made use of skills from local organisations (sustainable). Framework development was a reflective and iterative process although was described as lengthy and intermittent requiring further development (challenges).	A framework for promoting mental health was developed, including a range of indicators were defined; feeling safe, having a good place to live, having influence/feeling involved, feeling supported, feeling valued and respected, knowing where to get help, feeling hopeful. Measures for evaluating these indicators were also defined.
Cooke & Coggins [23]	Framework development of MWIA	To develop a toolkit enabling evaluation of Lewisham Neighbourhood Renewal Fund projects via a set of indicators/measures of mental wellbeing assessing how a particular policy promotes mental health.	Local indicators and stakeholders used. Indicator selection process highlighted differences between perspectives of policy makers, those delivering services and projects and the end users. Convincing funders of the value and importance of measuring mental wellbeing and developing new indicators identified as a challenge. Approach considered to be applicable to variety of different projects.	Framework development resulted in a structured MWIA toolkit. Progression included several stages and involved numerous stakeholders building on screening, literature review and indicator identification.
Holmes et al. [24]	MWIA was used to assess five community projects in the north-west of the UK.	To assess how “Target Wellbeing” projects impact the wellbeing of people living in disadvantaged communities.	Many of the templates from the MWIA toolkit were used for engagement of local community and organisations. Projects reported MWIA to be a positive process although some reported time limitations for indicator development. MWIA recommended as a community-dependent approach as opposed to finance (bid) dependent approach. Some younger age groups were more difficult to engage.	Results were summarised in terms of the MWIA protective pathways to mental wellbeing (i.e., Control, Resilience and Community Assets, Participation and Social Inclusion). Several project assessments resulted recommendations including multisector working, concrete actions such as repair work, need for participatory actions, training, funding and capacity building, increased mental health awareness.
Edmonds [25]	MWIA capacity building and assessment of community projects using MWIA framework	To build capacity by training up to 100 local people in the MWIA process and for them to carry out two MWIA’s in local organisations and establishing “learning networks” to sustain learning and development.	Structured training courses over 3 days with teamwork and mentor approach, all MWIA materials provided with budget for practical resources (e.g., venue, catering, crèche and travel). Some participants found concepts hard to understand and reported time restrictions. Process forged/strengthened partnership working and engagement although MWIA Teams being “external” to projects made it challenging to “sell” in certain areas. MWIA flexible approach highlighted as key.	Training completed for 93 individuals across several sectors meant increased capacity for delivering the MWIAs, strengthened local understanding of the evidence led mental health promotion, strengthened and new local partnerships, multi-agency working, integration of MWIA into local organisations and strategies.
West & Scott-Samuel A. [26]	MWIA assessment of community project using MWIA framework	To assess the potential impacts of the Liverpool 2008 European Capital of Culture programme on mental wellbeing, maximising positive effects and minimising negative impacts. Promoting health and wellbeing and reducing inequalities.	Mention of MWIA participatory nature and its awareness-raising role. An intensive assessment was chosen, and a range of stakeholders invited to half-day or full-day workshops. However, the time commitment required was highlighted as a restricting factor for full day workshops.	The findings demonstrated positive and negative impacts of the programme including recommendations around decision making, inequality, emotional wellbeing, neighbourhood safety, arts and culture, connectedness, physical health, engaging communities, physical environment, income, advocacy, communication, evaluation, sustainability.
Shearn [27]	MWIA assessment of community project using MWIA framework	To assess the impact of participation in interactive, inclusive gallery workshops at the Tate Modern for older inpatients and community service users. Identifying how ‘Art into Life’ impacts on the mental health and wellbeing of older adults with mental health problems and dementia.	Workshops led by mental health promotion officers. Engaged with participants in different manners to maximise communication. Close liaison and induction of new staff, including community staff. Consideration to collecting wellbeing measures as identified by the MWIA producing evidence for project evaluation which was appreciated.	Access to a ‘prestigious shared public space’ such as the Tate Modern was found to have significantly positive impacts on participants including interaction and engagement, empowering social inclusion, experience of interaction with public, participation, connectiveness and creating a sense of belonging.
Cooke et al. [28]	Description of actual MWIA toolkit using MWIA framework	To formulate the MWIA process in toolkit format, allowing for assessment of mental wellbeing impact of policies, services, programmes or projects in a wide range of settings and across all sectors.	Process outlined including flexible approach based on six-steps. Steps can be used in conjunction or ‘standalone’ manner. Process outlined in detail and all materials provided including case studies and different scenarios. The toolkit can be used by anyone with an interest in mental wellbeing impact assessment of policies, services, programmes or projects in a wide range of settings and across all sectors.	MWIA toolkit was finalised based on a six-step process including screening, scoping, appraisal, identification of potential positive or negative impacts, identification of indicators, recommendations and reporting.
Palinkas [29]	Development of conceptual framework using conceptual framework assessing mental health effects of oil spills	To develop a conceptual framework for understanding the mental health effects of oil spills to facilitate strategies for community resilience and targeted services to prevent and mitigate possible adverse effects.	The conceptual framework model offers a strategy for organising the evidence obtained from individual studies on mental health impact of oil spills and other technological disasters. The process is rather academic in nature summarising existing literature and emphasising on long term assessments making shorter term impact assessments less likely. Presumably also being a resource heavy process.	A conceptual framework based on a three-tiered approach including (1) environmental (2) community (3) intrapersonal aspects was outlined. The framework was deemed to have potential for informing further development and testing hypotheses specific to the mental health consequences of oil spills.
Todman et al. [30]	Pilot study assessing policy change using MHIA framework	Pilot study explored how MHIA can be used to empower residents to participate in reviewing a proposed housing policy change and ensure evidence-based policy decisions.	Screening of local indicators and workshops with community residents held—hosted by university. Training from a professional HIA organisation. A mayoral election slowed down the deliberative process.	The MHIA pilot demonstrated an effective means by which to engage community and other stakeholders in assessing the mental health implications of public decisions. The pilot provided a useful and informative foundation for a more extensive approach.
McDowell et al. [31]	Lessons learned and work in progress paper based on MHIA	To outline how MHIA ensures that mental health and health inequities are considered in decision making. Additionally, to highlight need for engaging community partners to evaluate and predict potential mental health outcomes of an employment policy.	Screening of local indicators and workshops with community residents held—hosted by university. Training from a professional HIA organisation. A mayoral election slowed down the deliberative process.	Important lessons learned on how and when to empower community members via strong relationships and commitment to a shared goal e.g improving school systems and increasing employability, connecting them in “fighting injustices that worsen health inequities”. Solidarity with community partners was considered critical for project success.
Ampuero et al. [32]	MWIA assessment of community event using MWIA framework	The objective was to use the MWIA framework to identify and describe the main factors enhancing mental wellbeing of people affected by a tsunami on Robinson Crusoe Island and their effects resilience.	The environmental model for mental wellbeing was intensive and academic in nature. Approx. 50 individual interviews lasting 30–90 min each and transcribed verbatim. Local newspapers, worksheets, writings, paintings and assessment and other qualitative information including field notes, photographs and video recording were used.	The main factors enhancing mental wellbeing in this context were: the natural environment, meaningful activities, local food, social activities, lifelong learning, transport and security. These factors influenced mental wellbeing through four main environmental dimensions: ecology, culture, milieu and organisation. They could influence mental wellbeing in a constant, multiple and cumulative way. Can be used to assist in the creation of community profiles and help generate indicators.
Burford et al. [33]	Assessment of MWIA use	To report on the strengths and weaknesses of using the MWIA in a workplace setting using two case studies.	Stakeholder interviews held. Interviewers had experience in facilitating full MWIA stakeholder workshops had been trained to delivery training for MWIA screening or were developers of the original MWIA. Interviews lasted 30–45 min, written notes were taken. Process dependent on organisational cooperation and reliant on engagement process. Results were reported as specific and not to be generalised, with susceptibility to observer bias.	MWIA was reported to be a useful tool both for teams to assess and discuss mental wellbeing in the workplace and as an intervention and diagnostic tool in its own right. Management committed was essential, giving responsibility back to teams although process may initially raise issues that lead to more stress.
Chiumento et al. [34]	Exploratory evaluation using MWIA framework	To evaluate and explore the Haven Green Space intervention on mental wellbeing of the children pre- and post-intervention and to assess the “Five Ways to Wellbeing” evaluation framework.	Small-scale mixed methods pilot studies conducted. MWIA adapted to children, e.g., workshops facilitated by research team shortened to 2 h pre and post intervention. MWIA used as a qualitative research tool, results triangulated against other qualitative and quantitative data. Analysed by two members of the research team.	The Haven Green Space intervention group-based socially interactive horticulture activities were associated with positive impacts upon the mental and emotional wellbeing of children experiencing behavioural, emotional and social difficulties. Further research is needed to verify this and to support using the “Five Ways” in intervention development and evaluation.
Stewart et al. [35]	MWIA assessment of community project using MWIA framework	To evaluate a Hub-based initiative for improving local mental health awareness, capacity building and co-ordination, assessing what components were influential, the impact of local circumstances and further actions.	Qualitative methods were used including focus groups and interviews to map each Hub’s activities alongside quantitative measures. Workshops with key stakeholders held in each region, facilitated by two members of the research team, audio-recorded. Collaboration with local “Hub leads” was instrumental for mentoring and building capacity of community members. Two Hub personnel participated in MWIA training. MWIA identified as a useful screening tool in identifying needs.	Hub sites built substantial community capacity to promote wellbeing, as measured by the Community Capacity Index. A total of 12 key considerations and critical success factors were identified, including opportunities to supplement individual capacity building strategies and a greater focus on the service coordination component of the Hub model moving forward.
Lin et al. [36]	Ecological framework development	To develop an understanding mental health risk pathways for children and adolescents in the context of COVID-19 pandemic.	The mental health impact of the COVID-19 pandemic on adolescents is approached via an ecological framework which is intended to develop integrative prevention and intervention strategies. An academic approach based on previous literature the ecological framework provides a theoretical ground for future measures should the similar pandemic occur in the future.	An ecological framework was outlined integrating cross-disciplinary strategies including microsystem, the mesosystem, the exosystem and the macrosystem.

## Data Availability

Not applicable.

## References

[1] Shim R., Koplan C., Langheim F.J.P., Manseau M.W., Powers R.A., Compton M.T. (2014). The Social Determinants of Mental Health: An Overview and Call to Action. Psychiatr. Ann..

[2] Silva M., Loureiro A., Cardoso G. (2016). Social Determinants of Mental Health: A Review of the Evidence. Eur. J. Psychiatry.

[3] Purtle J., Nelson K.L., Counts N.Z., Yudell M. (2020). Population-Based Approaches to Mental Health: History, Strategies, and Evidence. Annu. Rev. Public Health.

[4] Forsman A.K., Wahlbeck K., Aarø L.E., Alonso J., Barry M.M., Brunn M., Cardoso G., Cattan M., de Girolamo G., Eberhard-Gran M. (2015). Research Priorities for Public Mental Health in Europe: Recommendations of the ROAMER Project. Eur. J. Public Health.

[5] Wahlbeck K. (2015). Public Mental Health: The Time Is Ripe for Translation of Evidence into Practice. World Psychiatry.

[6] Huppert F.A., So T.T.C. (2013). Flourishing Across Europe: Application of a New Conceptual Framework for Defining Well-Being. Soc. Indic. Res..

[7] World Health Organization (2004). Promoting Mental Health: Concepts, Emerging Evidence, Practice: Summary Report.

[8] Galderisi S., Heinz A., Kastrup M., Beezhold J., Sartorius N. (2015). Toward a New Definition of Mental Health. World Psychiatry.

[9] Barry M.M. (2009). Addressing the Determinants of Positive Mental Health: Concepts, Evidence and Practice. Int. J. Ment. Health Promot..

[10] Topp C.W., Østergaard S.D., Søndergaard S., Bech P. (2015). The WHO-5 Well-Being Index: A Systematic Review of the Literature. Psychother. Psychosom..

[11] Tennant R., Hiller L., Fishwick R., Platt S., Joseph S., Weich S., Parkinson J., Secker J., Stewart-Brown S. (2007). The Warwick-Edinburgh Mental Well-Being Scale (WEMWBS): Development and UK Validation. Health Qual. Life Outcomes.

[12] International Association for Impact Assessment (IAIA) (2011). International Association for Impact Assessment (Online).

[13] Harris P., Sainsbury P., Kemp L. (2014). The Fit between Health Impact Assessment and Public Policy: Practice Meets Theory. Soc. Sci. Med..

[14] Harris P.J., Kemp L.A., Sainsbury P. (2012). The Essential Elements of Health Impact Assessment and Healthy Public Policy: A Qualitative Study of Practitioner Perspectives: Table 1. BMJ Open.

[15] Mindell J., Ison E., Joffe M. (2003). A Glossary for Health Impact Assessment. J. Epidemiol. Community Health.

[16] Winkler M.S., Furu P., Viliani F., Cave B., Divall M., Ramesh G., Harris-Roxas B., Knoblauch A.M. (2020). Current Global Health Impact Assessment Practice. Int. J. Environ. Res. Public. Health.

[17] Prince M., Patel V., Saxena S., Maj M., Maselko J., Phillips M.R., Rahman A. (2007). No Health without Mental Health. Lancet.

[18] Chanchitpricha C., Bond A. (2013). Conceptualising the Effectiveness of Impact Assessment Processes. Environ. Impact Assess. Rev..

[19] Keyes C.L.M. (2005). Mental Illness and/or Mental Health? Investigating Axioms of the Complete State Model of Health. J. Consult. Clin. Psychol..

[20] McGowan J., Sampson M., Salzwedel D.M., Cogo E., Foerster V., Lefebvre C. (2016). PRESS Peer Review of Electronic Search Strategies: 2015 Guideline Statement. J. Clin. Epidemiol..

[21] Popay J., Roberts H., Sowden A., Petticrew M., Araj L., Rodgers M., Britten N., Roen K., Duffy S. (2004). Guidance on the Conduct of Narrative Synthesis in Systematic Reviews. Draft Report from ESRC Methods Programme.

[22] Krasner E., Copeland J. (2004). Mental Health Impact of Neighbourhood Renewal Programmes. J. Ment. Health Promot..

[23] Cooke A., Coggins T. (2005). Neighbourhood Well-Being in Lewisham and Lambeth: The Development of a Mental Well-Being Impact Assessment and Indicator Toolkit. J. Public Ment. Health.

[24] Holmes L., West H., Dreaves H. (2009). Mental Well-Being Impact Assessment (MWIA) of Projects Funded by ‘Target: Wellbeing’. https://www.researchgate.net/publication/267234474_Mental_Well-being_Impact_Assessment_MWIA_of_Projects_funded_by_%27Targetwellbeing%27.

[25] Edmonds N. Well London—Mental Well-being Impact Assessment. http://www.welllondon.org.uk/39/mental-well-being-impact-assessment.html.

[26] West H.M., Scott-Samuel A. (2010). Creative Potential: Mental Well-Being Impact Assessment of the Liverpool 2008 European Capital of Culture Programme. Public Health.

[27] Shearn H. (2011). Mental Well-Being Impact Assessment.

[28] Cooke A., Friedli L., Coggins T., Edmons N., Michealson J., O’Hara K., Snowden L., Stansfield J., Steuer N., Scott-Samuel A. (2011). Mental Well-Being Impact Assessment; A Toolkit for Well-Being. https://healthycampuses.ca/wp-content/uploads/2014/07/MentalWellbeingImpactAssessmentAtoolkitforwellbe-1.pdf.

[29] Palinkas L.A. (2012). A Conceptual Framework for Understanding the Mental Health Impacts of Oil Spills: Lessons from the Exxon Valdez Oil Spill. Psychiatry.

[30] Todman L.C., Hricisak L.M., Fay J.E., Taylor J.S. (2012). Mental health impact assessment: Population mental health in Englewood, Chicago, Illinois, USA. Impact Assess. Proj. Apprais..

[31] McDowell T.L., Moore N., Holland J.N. (2015). Working Through Bound Liberation: A Community Engagement Framework for Health Partnerships. Prog. Community Health Partnersh. Res. Educ. Action.

[32] Ampuero D., Goldswosthy S., Delgado L.E., Miranda J.C. (2015). Using Mental Well-Being Impact Assessment to Understand Factors Influencing Well-Being after a Disaster. Impact Assess. Proj. Apprais..

[33] Burford C., Davey S., Knight A., King S., Cooke A., Coggins T. (2017). Mental Wellbeing Impact Assessment (MWIA) in the Workplace. J. Public Ment. Health.

[34] Chiumento A., Mukherjee I., Chandna J., Dutton C., Rahman A., Bristow K. (2018). A Haven of Green Space: Learning from a Pilot Pre-Post Evaluation of a School-Based Social and Therapeutic Horticulture Intervention with Children. BMC Public Health.

[35] Stewart V., Harris P., Betts H., Roennfeldt H., Wheeler A. (2019). Mental Health and Wellbeing Hubs Initiative: Evaluation Report. https://research-repository.griffith.edu.au/rest/bitstreams/1e39fb53-8443-4ba6-bedc-a29a99fd0cdd/retrieve.

[36] Lin P.-I., Srivastava G., Beckman L., Kim Y., Hallerbäck M., Barzman D., Sorter M., Eapen V. (2021). A Framework-Based Approach to Assessing Mental Health Impacts of the COVID-19 Pandemic on Children and Adolescents. Front. Psychiatry.

[37] Popay J., Whitehead M., Carr-Hill R., Dibben C., Dixon P., Halliday E., Nazroo J., Peart E., Povall S., Stafford M. (2015). The impact on health inequalities of approaches to community engagement in the New Deal for Communities regeneration initiative: A mixed-methods evaluation. Public Health Res..

[38] Birley M. (2003). Health Impact Assessment, Integration and Critical Appraisal. Impact Assess. Proj. Apprais..

[39] Quigley R.J., Taylor L.C. (2004). Evaluating Health Impact Assessment. Public Health.

[40] Kobau R., Seligman M.E.P., Peterson C., Diener E., Zack M.M., Chapman D., Thompson W. (2011). Mental Health Promotion in Public Health: Perspectives and Strategies from Positive Psychology. Am. J. Public Health.

[41] Botezat I., Campion J., Garcia-Cubillana P., Guðmundsdóttir D.G., Halliday W., Henderson N., Holte A., Santos M.J.H., Japing K., Katschnig H. (2017). Joint Action on Mental Health and Well-Being. Mental Health in All Policies.

[42] Bertotti M., Frostick C., Hutt P., Sohanpal R., Carnes D. (2018). A Realist Evaluation of Social Prescribing: An Exploration into the Context and Mechanisms Underpinning a Pathway Linking Primary Care with the Voluntary Sector. Prim. Health Care Res. Dev..

[43] Litwiller F., White C., Gallant K.A., Gilbert R., Hutchinson S., Hamilton-Hinch B., Lauckner H. (2017). The Benefits of Recreation for the Recovery and Social Inclusion of Individuals with Mental Illness: An Integrative Review. Leis. Sci..

[44] (2022). Mielenterveysvaikutusten Ennakkoarviointi: Kunnat.

[45] Craig P., Barr B., Baxter A.J., Brown H., Cheetham M., Gibson M., Katikireddi S.V., Moffatt S., Morris S., Munford L.A. (2022). Evaluation of the Mental Health Impacts of Universal Credit: Protocol for a Mixed Methods Study. BMJ Open.

[46] Wanjohi N.W., Harrison R., Harris-Roxas B. (2021). Health Impact Assessments of Health Sector Proposals: An Audit and Narrative Synthesis. Int. J. Environ. Res. Public. Health.

